# Reassessment of Long-Term Guideline-Directed Therapy for Peripheral Arterial Occlusive Disease in Primary Care

**DOI:** 10.7759/cureus.108553

**Published:** 2026-05-09

**Authors:** Joomong Park

**Affiliations:** 1 Medicine, Sacheon City Public Health Center, Sacheon, KOR

**Keywords:** antiplatelet therapy, intermittent claudication, peripheral arterial disease, primary care, secondary prevention, statin

## Abstract

Peripheral arterial occlusive disease is a manifestation of systemic atherosclerosis associated with increased risks of cardiovascular events, limb complications, and mortality. Guideline-directed medical therapy, including antiplatelet agents and statins, is recommended for secondary prevention, whereas cilostazol and exercise therapy are primarily intended for symptomatic relief of claudication. We report the case of a 68-year-old man with previously diagnosed peripheral arterial occlusive disease who presented to a primary care clinic with worsening intermittent claudication while receiving cilostazol without documented antiplatelet or statin therapy. His comorbidities included hypertension, type 2 diabetes mellitus with complications, chronic kidney disease, prior ischemic stroke, and former tobacco use. Physical examination revealed diminished pulses in the affected limb and a relatively cool foot without ulceration or acute infection. Laboratory evaluation showed serum creatinine 1.44 mg/dL (reference range: 0.70-1.30 mg/dL), estimated glomerular filtration rate 49 mL/min/1.73 m² (reference range: ≥60 mL/min/1.73 m²), and glycated hemoglobin 5.7% (reference range: 4.0%-5.6%). Aspirin 100 mg daily and rosuvastatin 10 mg daily were initiated for cardiovascular risk reduction. Cilostazol was later resumed for symptomatic management, and aspirin was changed to clopidogrel because of gastrointestinal intolerance. At the fourth week follow-up, the patient reported a modest increase in walking distance, although causality could not be established. This case highlights the importance of periodic medication reconciliation, reassessment of long-term vascular risk management, and recognition that symptomatic therapy should not substitute for guideline-directed secondary prevention in patients with established peripheral arterial occlusive disease.

## Introduction

Peripheral arterial occlusive disease is a common manifestation of systemic atherosclerosis and is associated with increased risks of myocardial infarction, stroke, limb loss, and death [1,2]. Contemporary guidelines recommend antiplatelet therapy, statin treatment, smoking cessation, exercise therapy, and aggressive risk factor modification for affected patients [1,2]. However, peripheral arterial disease remains underdiagnosed and undertreated in routine practice, and some patients do not receive appropriate secondary prevention [2,3].

Cilostazol is commonly used to improve walking distance and symptoms in patients with intermittent claudication, but it does not replace therapies intended to reduce long-term cardiovascular risk [1,2]. Primary care clinicians frequently encounter patients with chronic vascular disease whose medication regimens have evolved or whose prior records are incomplete. We report a case in which periodic medication review in primary care identified a gap in guideline-directed secondary prevention, prompting reassessment of long-term vascular risk management.

## Case presentation

A 68-year-old man presented to a primary care clinic with worsening exertional pain in the left lower extremity. He had previously been diagnosed with peripheral arterial occlusive disease at a tertiary hospital several years earlier, but detailed prior records were unavailable at the time of presentation.

For approximately three to four years, he had been taking cilostazol 100 mg daily for claudication symptoms. His medical history included hypertension, type 2 diabetes mellitus with complications, chronic kidney disease, and prior ischemic stroke. He was a former smoker with a 40-pack-year history.

He reported being able to walk only 10 to 15 meters before needing to stop because of pain. He denied rest pain, night pain, fever, or acute-onset limb symptoms. Based on these clinical features of exertional leg pain limiting walking distance of 10 to 15 meters without rest pain, ulceration, or tissue loss, his symptoms were consistent with Rutherford category 3 (severe claudication) and Fontaine stage IIb (claudication at less than 200 meters). As ankle-brachial index measurement was unavailable at the time of presentation, this classification is based on clinical criteria alone. Physical examination showed diminished pulses in the left lower extremity compared to the right side. The left foot was relatively cool. No ulceration, purulent discharge, or cellulitis was observed. There were no focal neurologic deficits, and the pain pattern was more consistent with vascular claudication than neurogenic claudication because symptoms were reproducibly related to walking distance rather than posture.

Baseline laboratory findings are summarized in Table 1. Glycemic control appeared relatively well maintained at the time of presentation despite his history of diabetes-related complications. Ankle-brachial index testing and vascular imaging were not available in the clinic at that visit. The patient presented without prior records from the tertiary hospital where he had originally been diagnosed. The diagnosis of peripheral arterial occlusive disease was therefore based on the established prior diagnosis reported by the patient, combined with consistent clinical findings, including diminished pulses and cool distal extremities on examination. The absence of objective hemodynamic confirmation is acknowledged as a limitation of this case.

**Table 1:** Baseline laboratory findings at presentation eGFR: Estimated glomerular filtration rate; HbA1c: Glycated hemoglobin

Parameter	Result	Reference range
Serum creatinine	1.44 mg/dL	0.70-1.30 mg/dL
Estimated glomerular filtration rate (eGFR)	49 mL/min/1.73 m²	≥60 mL/min/1.73 m²
Glycated hemoglobin (HbA1c)	5.7%	4.0%-5.6%

The serum creatinine of 1.44 mg/dL with an estimated glomerular filtration rate (eGFR) of 49 mL/min/1.73 m² corresponds to chronic kidney disease stage G3a, which represents an independent cardiovascular risk factor warranting careful consideration in treatment planning. Although the glycated hemoglobin (HbA1c) of 5.7% falls at the upper limit of the normoglycemic reference range, it should be interpreted in the context of his established history of type 2 diabetes with complications, suggesting relatively maintained glycemic control at the time of presentation rather than true normoglycemia.

Medication review revealed that he was not receiving antiplatelet therapy or statin therapy despite established vascular disease and multiple cardiovascular risk factors. Aspirin 100 mg daily and rosuvastatin 10 mg daily were started. Rosuvastatin 10 mg was selected as an initial moderate-intensity statin regimen, considering his age and chronic kidney disease, with plans for later titration according to tolerance and follow-up response. Cilostazol was temporarily withheld during reassessment and was resumed prior to the four-week follow-up visit for symptomatic treatment. Because of epigastric discomfort after aspirin initiation, aspirin was changed to clopidogrel 75 mg daily.

The patient was also advised to abstain from smoking, optimize foot care, and walk regularly, consisting of repeated symptom-limited walking with rest intervals several times daily as tolerated. A supervised exercise program was not locally available.

**Figure 1:**
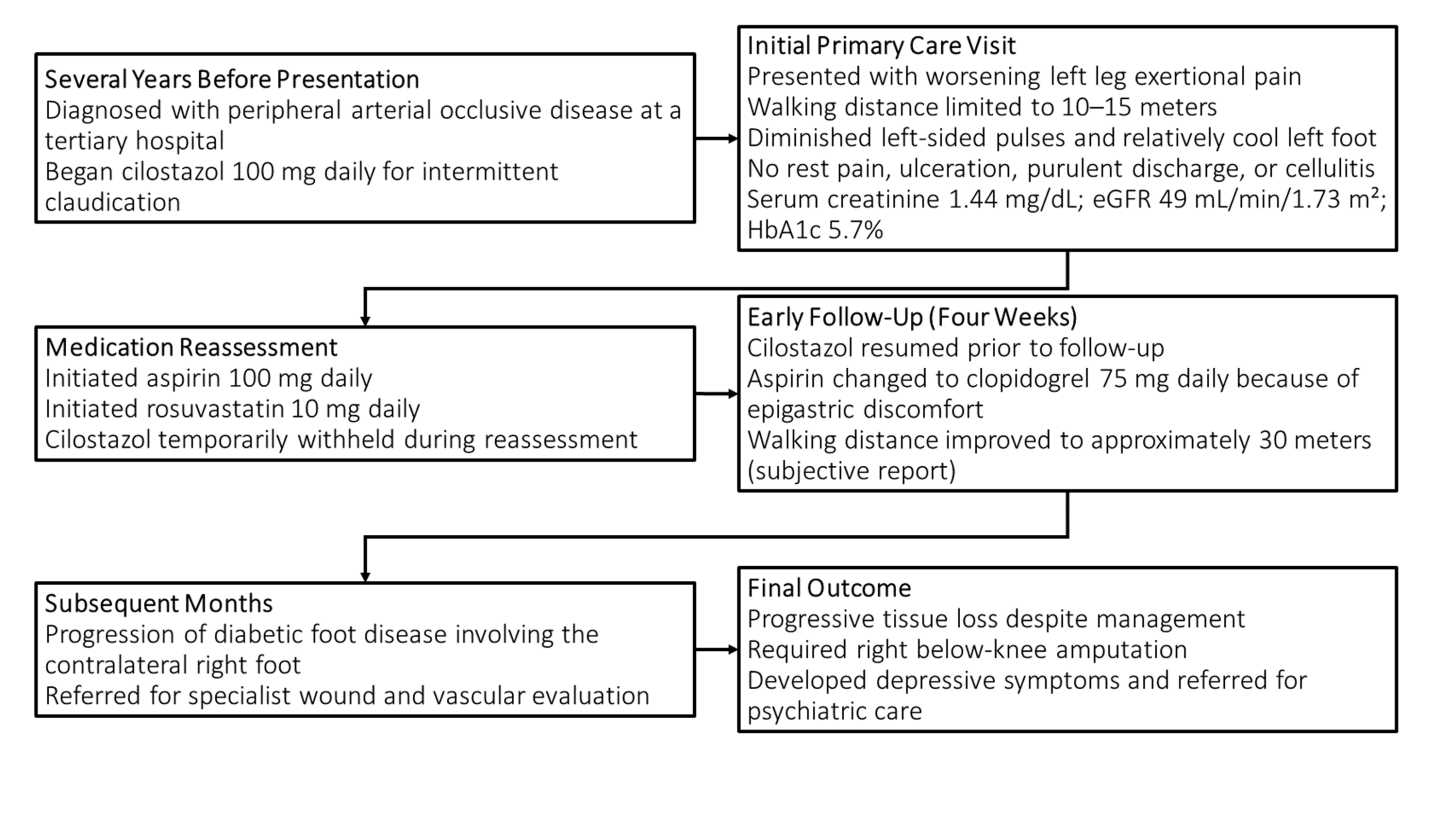
Clinical timeline of peripheral arterial occlusive disease management and contralateral limb progression PAOD: Peripheral arterial occlusive disease; eGFR: Estimated glomerular filtration rate; HbA1c: Glycated hemoglobin

At the fourth week follow-up, the patient reported an increase in walking distance to approximately 30 meters. Because no objective functional testing was performed, this subjective improvement was interpreted cautiously. Approximately three months after medication initiation, he developed progression of diabetic foot disease involving the contralateral right foot and was referred for specialist wound and vascular evaluation. Despite ongoing management, tissue loss progressed, and right below-knee amputation was performed approximately six months after the initial primary care visit. Following amputation, he experienced depressive symptoms related to functional decline and reduced independence. He was referred for psychiatric care and supportive follow-up. These symptoms may also have influenced later activity tolerance and self-reported functional status. The overall clinical course is summarized in Figure 1.

## Discussion

This case highlights the importance of reassessing long-term medical therapy in patients with established peripheral arterial occlusive disease, particularly when prior records are incomplete. Chronic medication regimens may persist for years without periodic review, and changes in record availability, treatment tolerability, adherence, or evolving guideline recommendations may create opportunities to reassess secondary prevention strategies.

Management of peripheral arterial disease requires distinction between therapies intended for symptom relief and therapies intended to reduce major adverse cardiovascular and limb events. Cilostazol may improve claudication symptoms and walking distance, whereas antiplatelet agents and statins are primarily used for risk reduction [1,2,4,5]. Clopidogrel has also demonstrated benefit over aspirin in certain high-risk vascular populations [6].

The reported improvement in walking distance should not be overinterpreted. Subjective improvement in walking distance does not constitute objective functional gain. Formal assessment tools, including ankle-brachial index measurement, treadmill testing, or six-minute walk testing, would be required to draw meaningful conclusions about treatment response to any specific therapeutic intervention. Aspirin is not expected to substantially improve claudication symptoms, and any benefit from statin therapy on walking performance would generally be gradual and limited in magnitude [7]. The observed change may have reflected reintroduction of cilostazol, behavioral changes, placebo effects, day-to-day variability, or altered motivation. Although a formal 'wound, ischemia, and foot infection' (WIfI) classification could not be assigned because hemodynamic testing was unavailable, the framework remains clinically relevant because it helps estimate amputation risk and guides the urgency of referral. In this patient, the absence of ulceration or overt infection would correspond to wound stage 0 and infection stage 0 within the WIfI framework. The ischemia stage could not be determined without hemodynamic testing. Severe exertional limitation and later progression of diabetic foot disease nevertheless support the importance of close surveillance and timely vascular reassessment.

Several factors may contribute to undertreatment, including fragmented transitions of care between tertiary and primary settings, limited clinician recognition of treatment gaps, therapeutic inertia, medication intolerance, nonadherence, polypharmacy, and limited access to vascular testing or supervised exercise programs [3,8]. In patients with severe symptoms, referral for vascular specialist evaluation and consideration of revascularization should also be considered.

Future primary care strategies may also incorporate digital health tools, including mobile reminders, wearable activity monitoring, and remote follow-up systems, to improve adherence, symptom tracking, and continuity of vascular risk management [9]. A major limitation of this case is the absence of ankle-brachial index measurement, structured walking assessment, and imaging correlation at presentation, which limits objective interpretation of disease severity and treatment response. This case also illustrates that peripheral arterial occlusive disease reflects systemic atherosclerotic burden; even when symptoms on one side appear stabilized, clinically significant disease in the contralateral limb may progress over time, reinforcing the need for ongoing whole-patient vascular surveillance.

## Conclusions

Periodic medication reconciliation in primary care can identify important gaps in secondary prevention among patients with established peripheral arterial occlusive disease. Symptom-directed therapy such as cilostazol should not substitute for antiplatelet therapy, statin treatment, and comprehensive cardiovascular risk reduction. Furthermore, objective reassessment and timely referral remain important when symptoms are severe, progressive, or discordant with current management.
